# Advances in Targeted and Systemic Therapy for Salivary Gland Carcinomas: Current Options and Future Directions

**DOI:** 10.3390/curroncol32040232

**Published:** 2025-04-16

**Authors:** Sushanth Sreenivasan, Rahim A. Jiwani, Richard White, Veli Bakalov, Ryan Moll, Joseph Liput, Larisa Greenberg

**Affiliations:** 1 Division of Internal Medicine, Allegheny Health Network, 320 East North Ave, Pittsburgh, PA 15212, USA; 2 Division of Medical Oncology, Allegheny Health Network, 314 East North Ave, Pittsburgh, PA 15212, USA; rahim.jiwani@ahn.org (R.A.J.); whiterj2@upmc.edu (R.W.); bakalov-veli@cooperhealth.edu (V.B.); ryan.moll@ahn.org (R.M.); joseph.liput@ahn.org (J.L.); larisa.greenberg@ahn.org (L.G.)

**Keywords:** salivary gland carcinoma, targeted therapy, head and neck tumors, immunotherapy, pleomorphic adenoma, chemotherapy

## Abstract

Salivary gland carcinomas (SGCs) represent a rare and heterogeneous group of malignancies accounting for 3–6% of all head and neck cancers. While surgical resection and radiotherapy remain the standard for locoregional control, systemic treatment is indicated for recurrent or metastatic disease. Advances in molecular profiling have identified actionable targets such as NTRK gene fusions, HER2, immune checkpoint regulators, androgen receptors, and RET receptors. These have facilitated the development of targeted therapies, including TRK inhibitors, HER2-directed agents, and androgen receptor modulators, as well as emerging combinations of immunotherapy and chemotherapy. Despite these advancements, challenges such as resistance mechanisms and limited therapeutic efficacy persist. Overall response rates remain relatively low across most systemic therapies, reflecting a persistent unmet clinical need. This review discusses the current landscape of treatment options and explores promising clinical trials and future directions to enhance outcomes for patients with SGCs.

## 1. Introduction

Salivary gland carcinomas (SGCs), while relatively rare, present a significant clinical challenge due to their histopathologic heterogeneity and variable treatment responses [1]. Tumors of the salivary gland have been increasing in incidence over the last 20 years [2], accounting for approximately 3–6% of all cancers of the head and neck today [3]. Recent advances in molecular profiling have unraveled key genetic and signaling pathways implicated in the pathogenesis of these tumors, thereby opening new avenues for precision medicine [4]. This review summarizes current research on targeted therapies in salivary gland malignancies, emphasizing how novel molecular insights are translating into tailored treatment strategies. We aim to highlight the potential and limitations of these targeted interventions.

## 2. Epidemiology

SGCs are rare, with an incidence rate of approximately 0.5–2 per 100,000 people per year [5,6,7]. These tumors can occur at any age but are commonly diagnosed in adults between the ages of 50 and 60 [8,9]. Geographically, incidence rates vary worldwide, with no specific worldwide distribution pattern identified [10]. Risk factors for developing salivary gland carcinomas include previous radiation exposure, occupational exposure to certain substances such as those in rubber manufacturing and asbestos, and genetic predispositions linked to specific hereditary syndromes such as BRCA1/2 mutations [11,12].

SGCs arise from major (parotid, submandibular, and sublingual) or minor salivary glands. Epidemiological studies reveal that while the majority of these lesions are benign—with parotid gland tumors constituting nearly 80% of benign cases—tumors arising in the submandibular and minor salivary glands exhibit a higher likelihood of malignancy [4]. Genetic mutations specific to each carcinoma subtype affect their biology and treatment paradigm. Moreover, variations in incidence based on demographic and geographic factors underscore the complexity of these neoplasms and the need for tailored diagnostic strategies [13].

SGCs exhibit a wide range of histological subtypes, each characterized by unique morphological features and clinical behaviors. Table 1 provides a detailed overview of the key histological subtypes, highlighting their morphology and clinical implications.

The French National Network on Rare Head and Neck Tumors (REFCOR) is a collaborative research platform that was established to address the challenges in managing rare head and neck cancers in France. In their comprehensive cancer registry, which contains more than 7200 patients, salivary gland tumors accounted for 32% of cases. The most frequent histological subtypes included adenocarcinoma not otherwise specified (13%), adenoid cystic carcinoma (12%), and mucoepidermoid carcinoma (9%) [14].

**Table 1:** Histological classification of salivary gland tumors.

Mucoepidermoid carcinoma (MEC)	The most common type of tumor, characterized by a mixture of mucus-secreting, epidermoid, and intermediate cells. It ranges from low to high grade.
Adenoid cystic carcinoma (AdCC)	Presents as a non-encapsulated well-circumscribed mass, which features cribriform, tubular, and solid growth patterns. It is known for its slow growth, high recurrence, and potential for distant metastasis.
Acinic cell carcinoma (ACC)	Typically, a low-grade tumor, cells can be granulated serous-type cells, primitive tubule cells, or undifferentiated polymorphous cells. Cell growth patterns include solid, papillary cystic, follicular, and microcystic.
Salivary duct carcinoma (SDC)	Resembles high-grade ductal carcinomas of the breast, histologically, and is aggressive with poor prognosis.
Polymorphous adenocarcinoma (PAC)	A low-grade tumor that usually occurs in the minor salivary glands. It shows diverse architectural patterns but uniform cytology.
Myoepithelial carcinoma	Rare, characterized by myoepithelial cell proliferation with varied architectural patterns and aggressive behavior
Epithelial–myoepithelial carcinoma (EMC)	An intermediate grade biphasic tumor with a combination of ductal and myoepithelial cells.
Clear cell carcinoma	An intermediate grade tumor, generally, which contains clear cells.
Basal cell adenocarcinoma	Generally low-grade tumor with basaloid appearance cells under the microscope.
Lymphoepithelial carcinoma	Major salivary gland consists of atypical lymphocytes with invasions into the adjacent ductal epithelium, lymphoepithelial lesions, and lymphoid follicles

Adapted from “Update from the 5th edition of the World Health Organization classification of head and neck tumors: Salivary glands” [15].

## 3. Pathogenesis

Salivary gland carcinomas (SGCs) encompass a diverse group of malignancies characterized by intra- and extracellular alterations, the dysregulation of signaling pathways, and aberrant receptor expression that collectively drive oncogenesis [16]. At the cellular level, mutations in oncogenes and tumor suppressor genes, such as TP53, HRAS, and PIK3CA, disrupt cell cycle control, apoptosis, and DNA repair mechanisms [17,18]. Extracellularly, changes in the tumor microenvironment, including stromal remodeling, angiogenesis, and immune evasion, play a critical role in tumor progression [19]. Aberrant expressions of growth factor receptors, such as HER2, EGFR, and c-KIT, lead to persistent activation of downstream signaling cascades, including the MAPK/ERK, PI3K/AKT, and JAK/STAT pathways [20]. These pathways promote proliferation, survival, and metastatic potential, while mechanisms like epithelial-to-mesenchymal transition (EMT) further facilitate tumor invasiveness [21]. Additionally, emerging evidence suggests that the interaction between cancer stem cells and their microenvironment contributes to therapy resistance and recurrence [22].

## 4. Diagnosis

Imaging modalities serve as the first-line, non-invasive approach for evaluating salivary gland tumors. These include ultrasound, computed tomography (CT), magnetic resonance imaging (MRI), and positron emission tomography (PET).

Ultrasound is the imaging modality of choice for the initial evaluation of superficial salivary gland lesions. It reliably differentiates solid from cystic components, assesses vascularity, and identifies intralesional characteristics such as calcifications and necrosis. In addition to its diagnostic utility, ultrasound provides real-time guidance for fine-needle aspiration (FNA), improving sampling precision and minimizing complications, especially in small, non-palpable, or anatomically complex lesions [23,24,25]. The incorporation of ultrasonography has significantly enhanced the diagnostic yield and safety of FNAC procedures, making it invaluable in modern salivary gland evaluation [25].

CT imaging is useful for evaluating tumor extent, bony invasion, and lymphadenopathy, which are essential for accurate staging [26]. MRI is the preferred modality for assessing tumor extent, soft tissue involvement, and perineural spread, especially in deep-seated tumors of the parotid, sublingual, and minor salivary glands [27].

Diffusion-weighted (DW) MRI with apparent diffusion coefficient (ADC) mapping further enhances diagnostic accuracy by quantifying water molecule diffusion within tissues. Malignant tumors, due to their high cellularity and dense structure, generally exhibit restricted diffusion and thus show low ADC values, while benign lesions tend to have higher ADC values. Apparent diffusion coefficient (ADC) mapping is a valuable noninvasive tool for differentiating tumor types prior to histopathological confirmation. Pleomorphic adenomas typically present with higher ADC values, whereas mucoepidermoid carcinomas and adenoid cystic carcinomas present with lower ones. Notably, non-Hodgkin malignant lymphoma (NHML) demonstrates very low ADC values, aiding in its differentiation from other malignancies with overlapping radiographic features [25,28].

PET imaging provides valuable insights into locoregional and distant metastases, and it has demonstrated superior accuracy to CT in assessing tumor extension, nodal involvement, local recurrence, and distant spread [29].

Ultimately, biopsy remains necessary for definitive histological diagnosis, regardless of whether a lesion is benign or malignant. Incisional biopsies may be appropriate for accessible, superficial lesions within the oral cavity, assuming no risk to vital structures such as nerves or blood vessels. However, for deeper sites like the parotid gland, fine-needle aspiration (FNA) is preferred due to the potential for nerve injury, particularly involving the facial nerve [30]. FNA offers a sensitivity of 80% and a specificity of 97% in distinguishing benign from malignant salivary gland neoplasms [31]. While FNA is minimally invasive, its diagnostic limitations stem from the limited preservation of tissue architecture, which may hinder accurate grading and subtyping of tumors. Core needle biopsy offers larger, architecturally intact tissue samples, improving accuracy in determining tumor subtype and grade [32].

Among FNA techniques, fine-needle aspiration cytology (FNAC) is generally favored over fine-needle aspiration biopsy (FNAB). FNAC utilizes cytological evaluation of aspirated cells, making it less invasive and associated with fewer complications. In contrast, FNAB involves tissue sampling and is formally contraindicated in many cases due to the higher risk of adverse events such as salivary fistula formation and needle tract tumor seeding [25,33]. FNAC, alternatively, offers excellent localization and targeting of lesions, reduces sampling error, and improves procedural safety, particularly in deep, small, or poorly accessible tumors [25,34].

## 5. Immunohistochemistry

While imaging and biopsy techniques are essential for the initial evaluation and classification of salivary gland tumors, further pathological analysis is required to determine tumor origin, biological behavior, and potential therapeutic targets. Immunohistochemistry (IHC) plays a critical role in this process by enabling the detection of specific cellular markers that aid in tumor characterization.

Immunohistochemistry (IHC) is a technique that leverages the specific binding affinity between antibodies and antigens to detect and localize target proteins within cells and tissues, which are then examined under a light microscope [35]. This method is used alongside traditional histopathological techniques to assess tissue morphology, identify cellular origin, and evaluate predictive and prognostic biomarkers [36]. By employing a panel of IHC stains, an IHC profile can be constructed to differentiate benign from malignant lesions and further characterize tumor subtypes. Listed in Table 2 are common IHC stains that are found in the corresponding salivary gland tumors.

## 6. Genome Sequencing

Genome sequencing is a technique used to determine the complete DNA sequence of an organism’s genome, providing comprehensive insights into its genetic composition [38]. The clinical application of this sequencing is tailored to capture specific clinically relevant biomarkers that can aid clinicians in diagnosing and tailoring treatments for patients [38]. With the development of targeted therapies and immunotherapy, these biomarkers have increased in importance in recent years. Listed in Table 3 are the common genomic alterations associated with their respective malignancies.

## 7. Treatment

The management of malignant salivary gland tumors requires a multidisciplinary approach tailored to tumor grade, location, involvement of local and distant structures, and stage. Surgery remains the cornerstone of treatment, often serving as the primary modality for localized disease. Postoperative radiotherapy is frequently employed in cases with high grade tumors or high-risk features such as positive margins, perineural invasion, or nodal metastasis to enhance local control. Conventional chemotherapy has historically played a limited role, primarily being used for palliation in advanced or recurrent diseases, though newer regimens are being explored. In recent years, immunotherapy has emerged as a promising option, particularly in tumors expressing actionable molecular targets.

### 7.1. Surgery

Surgical resection is the primary treatment for malignant salivary gland tumors and is often guided by the location, size, and histological subtype of the tumor [39]. Prognostic factors that can affect surgical outcomes include tumor grade, size, perineural spread, and lymphatic spread [39]. The main principle is complete resection with adequate margins and preservation of vital function [39].

Superficial parotidectomy is the preferred approach for tumors confined to the superficial lobe of the parotid gland, while a total parotidectomy is required for deeper or more invasive tumors [40]. The facial nerve courses through the parotid gland, so any manipulation of the gland risks damaging the nerve along with adjacent structures. Preservation of the facial nerve is a critical consideration, but in cases of extensive perineural invasion, nerve sacrifice with subsequent reconstruction may be necessary [41]. In submandibular and sublingual gland tumors, complete gland excision with adequate soft tissue margins is performed to minimize recurrence risk [42]. For high-grade malignancies or advanced-stage disease, neck dissection is recommended due to the risk of lymphatic spread, especially in adenoid cystic carcinoma and mucoepidermoid carcinoma [43]. Postoperative radiotherapy is frequently employed in cases with high grade tumors, positive margins, perineural invasion, or nodal involvement to improve local control [44].

### 7.2. Radiotherapy

Radiotherapy is essential in the management of salivary gland malignancies, particularly in cases with pathological features such as perineural invasion, positive surgical margins or lymph node metastasis [39]. Postoperative radiotherapy is often used as an adjuvant treatment post-surgery to improve local control and target any residual disease, especially in high-grade or locally advanced tumors [44]. According to the National Comprehensive Cancer Network guidelines, definitive or systemic RT is particularly useful for inoperable tumors that cannot be surgically removed due to their location or the patient’s overall health [43]. Intensity-modulated radiotherapy (IMRT) is the preferred technique, allowing precise delivery while minimizing exposure to surrounding structures such as the facial nerve, spinal cord, or brainstem [45]. Proton beam therapy and heavy ion therapy are emerging modalities that offer superior dose distribution, reducing toxicity and improving treatment tolerance [46]. A multimodal approach combining surgery and radiotherapy can enhance treatment outcomes, improve survival rates, and reduce the risk of recurrence. The RTOG 1008 trial is a phase II/III study evaluating the role of adjuvant chemoradiotherapy (CRT) with cisplatin vs. radiotherapy (RT) alone in patients with resected high-risk malignant salivary gland tumors. While the trial has completed accrual, the results are pending [47].

### 7.3. Chemotherapy

Chemotherapy plays a limited but important role in the treatment of salivary gland malignancies, primarily in cases of recurrent, metastatic, or inoperable disease [21,48]. The most commonly used regimen in the management of advanced salivary gland malignancies is the combination of cyclophosphamide, doxorubicin, and cisplatin (CAP) regimen [49]. A study by Dreyfuss et al. (1987) evaluated 13 patients with advanced salivary gland carcinomas treated with the CAP regimen, reporting an overall response rate of 46%, including both complete and partial responses [50]. The median duration of response was 5 months, indicating potential benefit in this patient population [51]. However, due to the heterogeneity of salivary gland malignancies, no universally accepted chemotherapy protocol exists, and treatment decisions are guided by histology, IHC, genome sequencing, and clinical presentation [52]. Table 4 presents the monotherapeutic agents tested on salivary gland malignancies, while Table 5 displays the combination of chemotherapy agents along with their objective response rates, median progression-free survival, and median overall survival as the key endpoint.

REFCOR recommendations reinforce this individualized approach, and consensus recommendations emphasize a tailored approach based on tumor subtype, stage, and molecular profile. For adenoid cystic carcinoma (ACC), which often demonstrates slow progression even at the metastatic stage, systemic therapy is generally not recommended in localized disease. Instead, active surveillance is often appropriate for metastatic presentations unless rapid progression is observed. Molecular screening is encouraged to identify potential targets for therapy [53].

In contrast, for non-ACC histologies such as salivary duct carcinoma and adenocarcinoma, which are more aggressive, early systemic therapy may be indicated. These cases benefit from molecular and immunohistochemical testing for biomarkers such as HER2 overexpression, androgen receptor positivity, and NTRK fusions, which can guide the selection of targeted therapies [54].

Chemotherapy remains a consideration primarily in advanced or inoperable disease, though no standardized protocol exists due to the heterogeneity of histologies [54]. As summarized in Table 4 and Table 5, while some combination regimens demonstrate modest activity, objective response rates remain low in many studies, often below 30%, underscoring the limited efficacy of conventional cytostatic approaches.

REFCOR highlights the importance of individualized treatment, biomarker-driven therapies, and clinical trial participation in the management of salivary gland malignancies.

**Table 4:** Single chemotherapy agent efficacy against salivary gland malignacies.

Study	Agent(s)	Target	Patients (n)	Subtype	Objective Response Rate (ORR)	Median Progression Free Survival (Months)	Median Overall Survival (Months)
Retro [52]	5-fluorouracil	Cytostatic drug	12	AdCC	4 (33%)		21
II [55]	Cisplatin	Cytostatic drug	10	AdCC	7 (70%)		
II [56]	Cisplatin	Cytostatic drug	25	Mixed Subtypes	ITT: 4 (16%)	7	14
II [57]	Cisplatin	Cytostatic drug	10	AdCC	0 (0%)	3	21
Retro [58]	Cisplatin	Cytostatic drugs	34	Mixed Subtypes	ITT: 13 (38%)	7	15
II [59]	Epirubicin	Cytostatic drug	20	AdCC	2 (10%)	3.7	15.6
Retro [60]	Methotrexate	Cytostatic drug	7	AdCC	0 (0%)		21
II [61]	Mitoxantrone	Cytostatic drug	32	AdCC	4 (13%)		18
II [62]	Mitoxantrone	Cytostatic drug	18	AdCC	1 (5%)		19

**Table 5:** Combination therapy efficacy against SGCs.

Phase of Study	Agents	Target	Pts	Subtype	Objective Response Rate (ORR)	Median Progression Free Survival (Months)	Median Overall Survival (Months)
II [63]	Carboplatin + Paclitaxel	Cytostatic drugs	14	AdCC:10, Non-AdCC:9	14%	6.0–13.5	12.5
Retro [64]	Carboplatin + Paclitaxel	Cytostatic drugs	38	AdCC:9, Non-AdCC:29	39%	6.5	26.5
II [65]	Cisplatin + 5-Fluorouracil	Cytostatic drugs	11	AdCC	0%	9.0	12.0
II [57]	Cisplatin + Doxorubicin + Bleomycin	Cytostatic drugs	9	AdCC	33%	10.0	12.0
Retro [50]	Cisplatin + Doxorubicin + Cyclophosphamide	Cytostatic drugs	13	AdCC:9, Non-AdCC:4	46%	6.5	12.0
Retro [66]	Cisplatin + Doxorubicin + Cyclophosphamide	Cytostatic drugs	8	AdCC:4, Non-AdCC:4	63%	5.0	11.0
II [67]	Cisplatin + Doxorubicin + Cyclophosphamide	Cytostatic drugs	22	AdCC:12, Non-AdCC:10	27%	-	21.0
II [68]	Cisplatin + Doxorubicin + Cyclophosphamide + 5-FU	Cytostatic drugs	16	AdCC:7, Non-AdCC:9	50%	-	16.8
II [69]	Cisplatin + Vinorelbine	Cytostatic drugs	16	AdCC:9, Non-AdCC:7	44%	7.0	11.0
II [70]	Cisplatin + Vinorelbine (First line)	Cytostatic drugs	42	AdCC:24, Non-AdCC:18	31%	6.0	10.0
II [70]	Cisplatin + Vinorelbine (Second line)	Cytostatic drugs	18	AdCC:10, Non-AdCC:8	6%	3.5	4.0
II [71]	Platin + Docetaxel	Cytostatic drugs	41	AdCC:26, Non-AdCC:15	46%	9.4	28.2
II [72]	Platin + Gemcitabine	Cytostatic drugs	33	AdCC:10, Non-AdCC:23	27%	13.8	-

AdCC: Adenoid cystic carcinoma.

### 7.4. Molecular and Immune-Based Therapeutics

**Figure 1:**
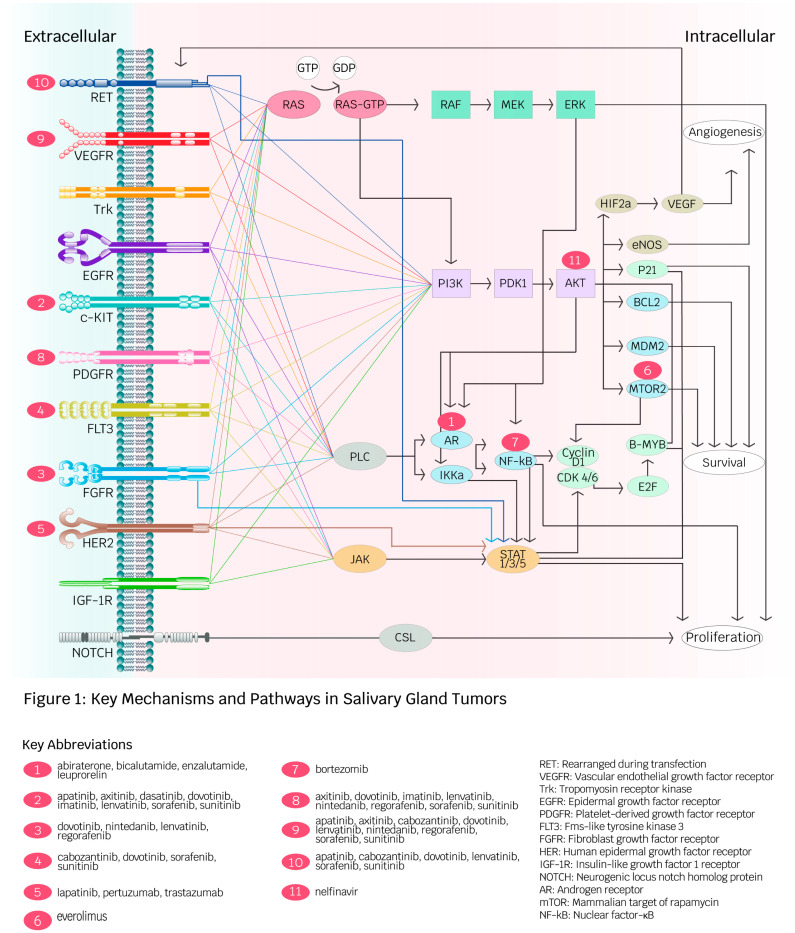
Key mechanisms and pathways in salivary gland tumors (created by Ambika Chaudry, used with permission). This diagram depicts receptors and intracellular pathways involved in angiogenesis, proliferation, and survival in salivary gland malignancies (SGMs). Red spheres illustrate targeted therapies developed for receptors and intracellular molecules. The diversity of pathways contributes to cancer development in SGMs and facilitates drug resistance against specific targeted therapies.

Figure 1 summarizes the key mechanisms and pathways of molecular and immune-based therapeutics discussed in the following sections.

#### 7.4.1. Neurotrophic Tropomyosin Receptor Kinases (NTRKs)

Neurotrophic tropomyosin receptor kinase (NTRK) gene fusions are oncogenic drivers in several cancers including salivary gland malignancies [73]. They are seen in up to 4% of salivary gland tumors, with the majority being mammary analogue secretory carcinomas (MASC) [74]. The most common tropomyosin receptor kinase fusion (TRK) found in SGC is ETV6-NTRK3, resulting from a translocation of chromosome 12 and 15. It leads to constitutive activation of the TRK pathway, promoting cellular proliferation and tumor growth [75]. Identification of the NTRK fusions in the cellular genome has become increasingly relevant due to the development of pan-TRK inhibitors. Larotrectinib and entrectinib are first generation pan-TRK inhibitors that result in the prevention of adenosine triphosphate, the energy storage molecule, from binding to the fusion protein, which prevents protein activations that normally lead to cell proliferation [76]. These inhibitors have demonstrated significant and durable responses across various tumor types in clinical trials, making them key players in the molecular approach to cancer treatment [77]. In a study evaluating larotrectinib in TRK fusion-positive salivary gland cancer, a 92% objective response rate (ORR) was observed, with 13% of patients achieving complete responses (CR) and 79% experiencing partial responses (PR) [78]. Pooled analysis of 153 TRK fusion-positive tumors’ response to larotrectinib, including 20 with salivary gland origin, showed an ORR of 90%, with median duration of response (DOR) of 35.2 months amongst this subpopulation [79]. Most reported treatment-related adverse events were grade 1–2 and included fatigue, elevated liver enzymes, nausea, and constipation [78].

Entrectinib has displayed efficacy in multiple phase I and II trials, and pooled analysis suggests efficacy, with overall response rates (ORR) of 86% and a manageable safety profile [80].

Unfortunately, as with other targeted agents, resistance develops to these agents over the course of treatment. Selitrectinib and repotrectinib, two selective TRK inhibitors are currently being investigated in the upfront and relapsed setting with the goal to overcome secondary resistance to first-line inhibitors [81,82]. Case reports have demonstrated promising activity of selitrectinib in heavily pretreated patients who develop resistance to first-line TRK inhibitors [83]. Table 6 lists some of the clinical trials that studied NTRK inhibitors and their efficacy, specifically their objective response rate, median progression-free survival, and median overall survival.

#### 7.4.2. HER2 Receptor

The HER2 receptor plays a significant role in the pathogenesis of salivary gland carcinomas, particularly in salivary duct carcinoma and mucoepidermoid carcinoma [85]. Human epidermal growth factor receptor 2 (HER2) is expressed on chromosome 17 (17q12), encoded by the ERBB2 oncogene, and its overexpression leads to aberrant cell proliferation and oncogenesis [86]. The HER2 protein activates downstream signaling cascades such as the PI3K/Akt and MAPK pathway, which leads to uncontrolled cellular growth [87].

HER2 overexpression in salivary gland carcinomas (SGCs) has been estimated at 43%, though rates vary across histological subtypes. Salivary duct carcinomas (SDCs) exhibit the highest HER2 positivity, with rates ranging from 45% to 83%, correlating with its aggressive clinical behavior [88,89]. Adenocarcinoma not-otherwise-specified (NOS) tumors demonstrate moderate expression with rates up to 38% [87].

Several trials utilizing HER2-directed therapies have shown clinical efficacy in SGC, and numerous trials are ongoing. A phase II trial from Japan of 57 patients with HER2-positive, locally advanced, recurrent, or metastatic salivary duct carcinomas showed an ORR of 70.2% and median OS of 39.7 months when using trastuzumab in combination with docetaxel. In this population, complete response (CR) was noted in 14% [90]. In a retrospective review, trastuzumab in combination with carboplatin and paclitaxel showed response in all patients, with a median DOR of 18 months [91]. Pertuzumab and trastuzumab combination has similarly shown efficacy in the MyPathway basket study, with an ORR of 60%, with 1 patient experiencing a CR [92]. As a second-line agent, ado-trasuzumab emtansine (TDM-1) has shown clinical response after progression on first-line taxane and trastuzumab, and further studies are warranted to better define the role of TDM-1 in SGC [93,94]. Table 7 lists the clinical trials that have studied HER2 inhibitors and their efficacy, specifically their objective response rate, median progression-free survival, and median overall survival. Common adverse effects include diarrhea, fatigue, nausea, elevated liver enzymes, and hematologic toxicities such as thrombocytopenia. In trials of antibody-drug conjugates, like T-DM1, infusion reactions, transaminitis, and cytopenias were observed [95].

#### 7.4.3. Immune Checkpoint Inhibitors

While immune checkpoint inhibitors (ICIs) have proven their effectiveness in head and neck cancers, their use has shown only modest benefit in salivary gland cancers. ICIs such as anti-PD1 (nivolumab, pembrolizumab) and anti-CTLA-4 (ipilimumab) block PD-1 or CTLA-4 from binding to T-cell receptors, thereby enabling them to recognize and target cancer cells [98,99]. However, responses vary significantly across histologic subtypes due to differences in the tumor immune microenvironment. Mucoepidermoid carcinoma (MEC) and salivary duct carcinoma (SDC) exhibit an immune-inflamed phenotype, making them more responsive to ICIs. Conversely, adenoid cystic carcinoma (ACC) is classified as an immune desert, characterized by low immune cell infiltration and poor ICI response [100].

A subset of PD-L1-positive (>1%) SGCs was evaluated in the KETNOTE-028 phase IB trial, utilizing pembrolizumab. Results showed ORR of 12% at median follow-up of 20 months; however, median DOR was just 4 months [101]. Similarly, subgroup analysis of KEYNOTE-158, which evaluated the efficacy of pembrolizumab in mismatch repair deficient non-colorectal cancers, showed a partial response in 1 of 3 SGC patients with tumor mutation burden (TMB) > 10 mut/Mb [102]. A multicenter retrospective study evaluating the safety and efficacy of nivolumab in 24 SGC patients who received prior chemotherapy conferred similar results, with ORR of 4.2%, median progression-free survival (PFS) of 1.6, and median OS of 10.7 months. Interestingly, one patient continued nivolumab for 28 months without progression of disease (POD), indicating the possibility of durable response in some patients [103]. Among trials studying immunotherapy, objective response rates are below 15%, highlighting the immune-evasive nature of salivary gland malignancies.

Clinical trials evaluating the combination of immunotherapy with chemotherapy or other novel agents, to overcome its poor efficacy, in SGC are ongoing. Vorinostat, a histone deacetylase inhibitor, in combination with pembrolizumab displayed median PFS and OS of 6.9 and 14 months, respectively, in 25 patients with recurrent metastatic SGC [104]. Recruitment is ongoing for clinical trials investigating pembrolizumab in combination with pemetrexed and lenvatinib with pembrolizumab in recurrent/metastatic salivary gland cancer [105,106]. The combination of the cytotoxic T-lymphocyte-associated protein 4 inhibitor ipilimumab plus nivolumab is being investigated in an ongoing clinical trial in recurrent or metastatic SGC, with results expected in May 2025 [107].

Immune checkpoint inhibitors (ICIs), particularly anti-PD-1 and anti-PD-L1 agents, are associated with a range of immune-related adverse events (irAEs); xerostomia (dry mouth) due to salivary gland hypofunction, oral lichenoid reactions, and pemphigoid-like lesions are among the most notable toxicities [108]. Table 8 lists the clinical trials that have studied immune checkpoint inhibitors and their efficacy, specifically their objective response rate, median progression-free survival, and median overall survival

#### 7.4.4. Androgen Receptors

Androgen receptor (AR) positivity is a frequently identified and unique feature of SGC and has altered the treatment landscape in recent years. AR positivity varies based on histology, with reported incidence in 86% of salivary duct carcinomas, 26% of NOS, and 15% of acinic cell carcinomas, with less frequent incidence in mucoepidermoid carcinoma (5%) and adenoid cystic carcinoma (5%) [110].

Therapeutic targeting of AR has been studied in the first-line metastatic setting for recurrent SGC and in the adjuvant setting. A Dutch study of 35 AR-positive metastatic SGC patients treated with first-line androgen deprivation therapy (ADT) showed a clinical benefit ratio of 50%, with a median OS of 17 months compared to 5 months in 43 patients treated with best supportive care [111]. In a retrospective review by Locatty et al., 17 patients with AR-positive recurrent/metastatic salivary gland cancer treated with androgen deprivation therapy (ADT) displayed an ORR of 64.7%, 3-year PFS of 11.8%, and 5-year OS of 19.3% [112]. A study in patients with stage 4A SGC compared adjuvant androgen deprivation therapy (ADT) following primary resection to no adjuvant therapy. The results showed a significant improvement in 3-year disease-free survival (DFS), increasing from 27.7% in the control group to 48.2% in the ADT group (*p* = 0.037) [113]. A Japanese study showed leuprorelin acetate in combination with bicalutamide to be both well tolerated and efficacious in AR-positive metastatic or locally advance unresectable SGC, with median PFS of 8.8 months, OS of 30.5 months, and clinical benefit rate of 75.0% [114]. In patients with AR-positive salivary gland tumors, treatment with ADT warrants consideration.

Despite the promising results of targeting the androgen pathway as a therapeutic option, hormone resistance will eventually pose an obstacle. Initial results of second-line therapy with enzalutamide failed to meet its primary endpoint, showing only 2 out of 46 patients with an objective response [115]. Commonly observed adverse effects included fatigue, hypertension, hot flashes, and weight loss. While the treatment was generally well tolerated, grade 3 or higher adverse events occurred in 35.5% of patients, including skin rash, anemia, and leukopenia [116]. A single-institution phase II trial evaluating abiraterone acetate as a second-line therapy in ADT-resistant, AR-positive SGC included 23 patients, with an ORR of 21% and a disease control rate of 62.5%. The median DOR was 5.8 months with a PFS of 3.7 months and a median OS of 22.4 months [117]. Numerous ongoing trials are evaluating ADT combined with other modalities, such as the currently enrolling multicenter phase II trial examining the combination of goserelin acetate with pembrolizumab, with results expected in 2025 [118]. Table 9 summarizes the results of the aforementioned trials.

#### 7.4.5. RET Fusion Gene

The RET fusion gene serves as an oncogenic driver in certain salivary gland tumors, particularly secretory carcinomas. It fuses with ETV6 and NCOA4, forming a fusion oncoprotein that promotes tumorigenesis by activating the RET tyrosine kinase receptor [109]. The resulting fusion oncoprotein activates RET, leading to uncontrolled cellular proliferation via the RAS-RAF-MEK-ERK pathway and enhanced survival and resistance to apoptosis through the PI3K-AKT-mTOR pathway [120,121].

Rearranged during transfection (*RET*) fusions are identified in less than 1–2% of salivary gland cancers (SGCs) [122]. Pan-tyrosine kinase inhibitors have been studied against salivary gland malignancies with modest overall response rates; a few studies have been tabulated in Table 10. These have broader kinase activity but may be less effective and more toxic compared to selective RET inhibitors [121]. Targeting the RET fusions potentially could add to our armamentarium against salivary gland malignancies. Selpercatinib, a highly active anti-RET kinase inhibitor, received tumor agnostic approval after the Libretto trial showed antitumor activity across a variety of histopathologies [123]. In this trial, 2 of 4 patients with RET fusion-positive SGC were found to exhibit an objective and durable response, with 1 patient achieving a complete response by independent review committee [123]. Selective RET inhibitors exhibit mucocutaneous adverse events (MAEs), which include xerostomia (37%), skin rash (18%), periorbital edema (12%), and xerosis (9%) [124]. Nearly half of patients may experience more than one MAE, and while the vast majority are grade 1–2, approximately 14% of patients required dose interruption or modification due to these events [124]. Table 11 summarizes the clinical trial efficacy of selective RET kinase inhibitors.

#### 7.4.6. BRAF Gene; BRAF V600E Mutation

The BRAF gene is a component of the RAS-RAF-MEK-ERK signaling pathway, which plays a crucial role in cellular proliferation and differentiation [130]. The BRAF V600E mutation causes RAS-independent activation of the MAPK pathway. The mutated kinase is constitutively active and phosphorylates MEK, leading to persistent ERK activation and uncontrolled cellular proliferation [131]. It also suppresses pro-apoptotic signals by altering the balance of the bcl-2 family of proteins leading to tumor cellular survival [132].

BRAF mutations, particularly BRAF V600E, are well-recognized oncogenic drivers in melanoma, thyroid cancer, and colorectal cancer but are rarely observed in salivary gland malignancies [133,134]. The exact incidence of the BRAF V600E mutation in salivary gland carcinomas (SGCs) is unknown. One analysis of 65 patients with SGCs did not find any cases of BRAF V600E mutation [135]. However, salivary duct carcinoma, a subtype of SGC, has occasionally been reported to harbor BRAF V600E mutations [136]. Molecular profiling is essential in SGC to identify patients who may benefit from targeted therapy.

Dabrafenib and trametinib are FDA-approved potent inhibitors of the proto-oncogenes *BRAF* and *MEK*, indicated for patients with BRAF V600E-mutated solid tumors who have progressed on prior therapy or lack alternative treatment options [137]. As molecular profiling becomes more common, combination therapy should be considered if an actionable mutation is identified, based on available data. The combination has a distinct toxicity profile that includes pyrexia, fatigue, nausea, vomiting, skin rash, diarrhea, and elevated liver enzymes. Cutaneous reactions (including acneiform eruptions and alopecia), ocular disturbances, and transaminase elevations are also reported [137].

Table 12 describes the efficacy of a dabrafenib + trametinib regimen and its overall response rate, median progression-free survival, and median overall response.

#### 7.4.7. C-Kit Receptor

Another promising therapeutic target in salivary gland malignancies is the c-KIT receptor, a tyrosine kinase receptor encoded by the *KIT* gene. Activation of this receptor plays a critical role in malignant processes such as tumor invasion and survival [139,140].

C-KIT overexpression is highly expressed in 80–90% of cases of adenoid cystic carcinoma (AdCC), lymphoepithelioma-like carcinoma, and myoepithelial carcinoma. It serves as a hallmark marker of this cancer subtype [140]. Given its significant role in tumor biology, c-KIT has been explored as a potential therapeutic target in clinical trials. However, clinical outcomes have been disappointing. Studies utilizing imatinib, a c-KIT inhibitor, in c-KIT-positive AdCC reported an objective response rate (ORR) of 0% in several trials, with median progression-free survival (PFS) ranging between 2.3 and 6 months [141,142]. Combining tyrosine kinase inhibitors with other therapies, such as cisplatin, has shown modest improvements, achieving an ORR of 11% and a PFS of 15 months [143]. Imatinib is well tolerated; however, patients may experience serious but less common toxicities such as hepatotoxicity, cytopenia, and cardiac events such as QT prolongation [144].

Table 13 describes the efficacy of imatinib in adenoid cystic carcinoma in clinical trials, specifically, its overall response rate, median progression-free survival, and median overall response.

These suboptimal outcomes can be attributed to mechanisms of resistance, including the activation of alternative, redundant signaling pathways such as JAK, RAS, or PI3K pathways, genomic heterogeneity, and the tumor’s ability to bypass single-receptor inhibition [21]. Tyrosine kinase inhibitors have demonstrated drug-specific resistance and resistance that develops from the tumor microenvironment, limiting their long-term efficacy [145,146]. Some resistance mechanisms include secondary mutations in targeted kinases, activation of compensatory signaling pathways, epigenetic or transcriptional adaptations, and tumor microenvironment adaptations that enable tumor cells to evade treatment [145,146]. These factors underscore the importance of continuing research into next-generation tyrosine kinase inhibitors and combination therapies, which may be required to exploit c-KIT as a therapeutic target in salivary gland malignancies.

#### 7.4.8. VEGF Pathway

The vascular endothelial growth factor (VEGF), specifically VEGFR 1 and VEGFR 2, plays a critical role in angiogenesis, tumor growth, and metastasis in salivary gland carcinomas (SGCs) [147]. This overexpression has led to clinical trials exploring VEGF inhibitors as potential therapeutic agents.

Clinical trials have evaluated various tyrosine kinase inhibitors (TKIs), demonstrating variable efficacy depending on the specific agent and tumor subtype. Table 14 summarizes the efficacy of different TKIs tested in clinical trials. A phase II clinical trial of rivoceranib in 65 patients with adenoid cystic carcinoma (AdCC) reported an overall response rate (ORR) of 46% and a median progression-free survival (PFS) of 19.7 months [148]. Similarly, axitinib, tested in multiple phase II trials, showed an ORR ranging from 0% to 17%, with PFS between 5.5 and 14.5 months [149,150]. In contrast, lenvatinib demonstrated an objective response rate of 16% and a PFS of up to 17.5 months in a phase II trial [106].

The VEGF pathway remains a critical area of research, particularly in understanding resistance mechanisms and identifying patient subgroups who may benefit from targeted therapies. Newer TKIs such as nintedanib, a triple-receptor TKI, and rivoceranib, a VEGFR2 inhibitor, have shown promising results. In a phase II trial of nintedanib for recurrent or metastatic salivary gland cancer, the study reported a disease control rate (DCR) of 75%, with a 6-month PFS rate of 60% [151]. Similarly, in a phase II trial of rivoceranib, the drug demonstrated an ORR of 15.1% and a median PFS of 9 months in patients with recurrent or metastatic adenoid cystic carcinoma (ACC) [152]. Nintedanib is associated with gastrointestinal adverse events, diarrhea, nausea, decreased appetite, and notably elevated liver enzymes [153]. Rivoceranib, a highly selective VEGFR2 inhibitor, has a tolerable safety profile. It is associated with grade >3 hypertension, anorexia, and diarrhea [154].

**Table 14:** Efficacy of tyrosine kinase inhibitors in VEGF+ SGMs in clinical trials.

Phase	Agent	Target	Pts (n)	Subtype	Objective Response Rate	Median Progression-Free Survival (Months)	Median Overall Survival (Months)
II [148]	Rivoceranib	VEGFR, RET, c-KIT	65	AdCC	30 (46)	19.7	Not reported
II [150]	Axitinib	VEGFR, PDGFR, c-KIT	27	AdCC	0%	10.8	NR
II [150]	Axitinib (after cross-over)	VEGFR, PDGFR, c-KIT	26	AdCC	3 (12)	14.5	27.2
II [152]	Rivoceranib	VEGFR2	61	Recurrent/metastatic adenoid cystic carcinoma (AdCC)	15.1%	9 months	Not Reported

ORR: Overall response rate, MPFS: Median progression-free survival, MOS: Median overall survival in months, AdCC: Adenoid cystic carcinoma.

#### 7.4.9. PI3K/Akt Receptor

The PI3K/Akt pathway is a key signaling pathway in cell proliferation, metabolism, and survival, and its dysregulation has been implicated in oncogenesis [155]. This pathway is activated through upstream signals such as EGFR (epidermal growth factor receptor), and an increase in Akt phosphorylation promotes tumor cell growth, survival, and angiogenesis [156]. Aberrant activation of this pathway occurs through mutations in the PIK3CA gene, PTEN loss, or Akt amplification, leading to enhanced tumor growth and resistance to apoptosis [157]. The PI3K/Akt pathway has been actively investigated as a therapeutic target for cancer treatment. mTOR inhibitors, namely everolimus and tesirolimus, were the first PI3K pathway-targeted drugs, demonstrating limited single-agent efficacy in head and neck cancers, including salivary gland malignancies [158]. Table 15 lists the overall response, median progression-free survival, and median overall survival of nelfinavir and everolimus in clinical trials [159,160]. The overall response rate of both agents was 0 in clinical trials, which is not promising [159,160]. Newer agents, such as dual PI3K/mTOR inhibitors and isoform-selective PI3K inhibitors, have been developed to enhance pathway inhibition while reducing toxicity. In a phase I dose-escalation study of MSC2363318A, a dual p70S6K/Akt inhibitor, patients with advanced malignancies, including adenoid cystic carcinoma of the salivary glands, achieved stable disease (SD) for over 36 weeks [161]. Clinical trials evaluating PI3K pathway inhibitors as monotherapies have yielded limited success, prompting increased interest in combination strategies with immune checkpoint inhibitors, chemotherapy, or other targeted therapies to enhance treatment efficacy [162]. PI3K/Akt pathway inhibitors are associated with class-specific adverse effects, including hyperglycemia, hepatotoxicity, non-infectious pneumonitis, cutaneous toxicities (e.g., rash, pruritus), mucositis, and immunosuppression. These toxicities are primarily due to the on-target effects of the inhibitors on their respective physiological signaling pathways [163].

## 8. Conclusions

The evolution of cancer treatment towards precision medicine underscores the critical role of molecular profiling, specifically through next-generation sequencing. As molecular diagnostics become integrated into routine clinical practice, the application of targeted therapies tailored to specific genetic and molecular aberrations is anticipated to expand treatment options and enhance clinical outcomes. Ongoing research and clinical validation remain essential to fully harness the potential of personalized therapeutic strategies in this challenging subset of head and neck malignancies. Nonetheless, response rates across most available therapies remain low, emphasizing the urgency of continued translational research and clinical trial enrollment to identify more effective, durable treatment options.

## Figures and Tables

**Table 2:** Immunohistochemistry signature of salivary gland tumors.

Tumor	Immunohistochemistry
Polymorphous adenocarcinoma	p63, p53, CKAE 1/3, Vimentin, S100
Pleomorphic adenoma	CK7, CAM5, SMA, C-kit, SMMHC, Calponin +PLAG1
Adenoid cystic carcinoma	+p63, +P40, CD117, CK7, CKAE1/3, EMA, DOG1, AR, CEA
Mucoepidermoid carcinoma	+CK7, +P63, EMA, CK20, S100, SMA, SMMHC, Calponin
Clear cells	+P63, -CD10
Myoepithelial carcinoma	+ CK7, HMWK, CKAE1/3, EMA, +Vimentin, +S100, +SMA, SMMHC
Acinic cell carcinoma	CK7, DOG1
Salivary ductal carcinoma	AR +, Her2+, CK7, CAKE1/3, EMA, GCDFP15, CEA

Table adapted from: [37].

**Table 3:** Molecular alterations in salivary gland tumors.

Tumor	Molecular Alteration
Adenoid cystic carcinoma	NOTCH1 mutation, EGFR, KIT overexpression, MYB-NF1B fusion gene
Mucoepidermoid	PI3KCA, BRCA1/2, CDKN2A mutations, CRTC1-MAML2 fusion gene
Salivary duct carcinoma	ERBB2 (HER2), AR
Acinic cell carcinoma	CDKN2A+, PPP1R13B deletion, NR4A3 rearrangement
Pleomorphic adenoma	HER-2 overexpression, ERBB2 amplification, HRAS mutation, PI3KCA mutation; PTEN loss and PLAG1 rearrangements
Secretory carcinoma	PRSS1, MLH1, MUTYH and STK11 mutation, ETV6-NTRK3 fusion gene present
Intraductal carcinoma	KRAS and/or PI3KCA mutation, NCOA4-RET fusion gene
Myoepithelial carcinoma	KRAS and HRAS mutation, SMARCB1 deletion

Table cited from [39].

**Table 6:** Clinical trials measuring neurotrophic tropomyosin receptor kinase inhibitor efficacy.

Phase of the Trial	Agent	Target Molecule	Patients	Subtype of Tumor	Objective Response Rate	Median Progression-Free Survival (Months)	Median Overall Survival (Months)
II [78]	Larotrectinib	NTRK1/2/3	24	TRK fusion-positive salivary gland cancers	92% (95% CI: 73–99)	78% at 24 months	Not reported
I/II [84]	Entrectinib	NTRK1/2/3, ROS1	150 (Various tumors)	Multiple solid tumors	61.3% (95% CI: 53.1–69.2)	13.8 months (95% CI: 10.1–20.0)	37.1 months (95% CI: 27.2-NE)
II [80]	Entrectinib	NTRK, ROS1, ALK	10	NTRK fusion-positive AdCC and non-AdCC	86%	Not reported	Not reported

MEC: Mucoepidermoid carcinoma, SDC: Salivary ductal carcinoma, AdCC: Adenoid cystic carcinoma.

**Table 7:** Clinical trials measuring HER2 inhibitor efficacy.

Phase	Agent	Target	Pts(n)	Subtype	Objective Response Rate	Median Progression-Free Survival (Months)	Median Overall Survival (Months)
II [96]	Trastuzumab	HER2	14	HER2+ AdCC and Non-AdCC, including MEC, adeno, and SCC	8%	4.2	-
II [92]	Trastuzumab + Pertuzumab	HER2	16	HER2+ non-AdCC, including MEC, SDC, and adeno	56%	9.1	20.4
II [90]	Trastuzumab + Docetaxel	HER2	57	HER2+ SDC	70%	8.9	39.7
II [97]	Ado-trastuzumab emtansine	HER2-targeted ADC	10	HER2+	90%	Not reached	Not reached

MPFS: Median progression-free survival, MOS: Median overall survival, MEC: Mucoepidermoid carcinoma, SDC: Salivary ductal carcinoma, AdCC: Adenoid cystic carcinoma.

**Table 8:** Clinical trials evaluating immune checkpoint inhibitor efficacy.

Phase	Study	Agent	Target	Subtype	Patient	Objective Response Rate	Median Progression-Free Survival (Months)	Median Overall Survival (Months)
Ib [101]	KEYNOTE-028	Pembrolizumab	PD-1	26	PD-L1+ AdCC and non-AdCC	12%	4	13
II [102]	KEYNOTE-158	Pembrolizumab	PD-1	109	AdCC and non-AdCC, including MEC, SDC, AcCC, etc.	5%	4	21.1
II [104]	Pembrolizumab + Vorinostat	Pembrolizumab + Vorinostat	PD-1 + Histone deacetylase	25	AdCC and non-AdCC, including MEC, AcCC, and other subtypes	16%	6.9	14
II [109]	Pembrolizumab + IMRT	Pembrolizumab + IMRT	PD-1	10	AdCC	0%	4.5	Not reached

ORR: Overall response rate MPFS: Median progression-free survival, MOS: Median overall survival in months, AdCC: Adenoid cystic carcinoma, MEC: Mucoepidermoid carcinoma, SDC: Salivary ductal carcinoma.

**Table 9:** Clinical trials measuring androgen receptor efficacy.

Phase	Agent	Target	Pts (n)	Subtype	Objective Response Rate	Median Progression-Free Survival (Months)	Median Overall Survival (Months)
II [119]	Enzalutamide	AR	46	AR+ AdCC and non-AdCC, including SDC and ex pleomorphic adenoma	15%	5.6	17
II [117]	Abiraterone acetate	CYP17A1	24	AR+ non-AdCC, including SDC and adeno	21%	3.7 (SDC: 4.0, Adeno: 2.5)	22.5 (SDC: Not reached, Adeno: 8.8)
II [115]	Leuprorelin acetate + Bicalutamide	GnRH receptor agonist + AR	36	AR+ non-AdCC, including SDC and adeno	42%	8.8	30.5

ORR: Overall response rate. MPFS: Median progression-free survival, MOS: Median overall survival in months, AdCC: Adenoid cystic carcinoma.

**Table 10:** Pan-tyrosine kinase inhibitor efficacy against SGM.

Phase	Agent	Target	Pts (n)	Subtype	Objective Response Rate	Median Progression-Free Survival (Months)	Median Overall Survival (Months)
II [125]	Cabozantinib	MET, RET, AXL, VEGFR2, FLT3, c-KIT	21	AdCC and non-AdCC, including MEC, SDC, and others	10%	9.4 (AdCC)/7.2 (Non-AdCC)	27.5 (AdCC)/14.2 (Non-AdCC)
II [106]	Lenvatinib	VEGFR, FGFR, PDGFR, RET, KIT	32	AdCC	16%	17.5	27
II [126]	Regorafenib	VEGFR, FGFR, PDGFR	38	AdCC	0%	Not reported	Not reported
II [127]	Dovitinib	VEGFR, c-KIT, PDGFR, CSF-1R, RET, TrkA, FLT3	32	AdCC	3%	6	20.6

ORR: Overall response rate, MPFS: Median progression-free survival, MOS: Median overall survival in months, MEC: Mucoepidermoid carcinoma, SDC: Salivary ductal carcinoma, AdCC: Adenoid cystic carcinoma.

**Table 11:** Selective RET inhibitor efficacy against various solid malignancies.

Phase	Agent	Target	Pts (n)	Subtype	Objective Response Rate	Median Progression-Free Survival (Months)	Median Overall Survival (Months)
I/II [128]	Selpercatinib	RET	316	NSCLC	61% (pretreated); 84% (treatment-naive)	24.9 months (pretreated); 22.0 months (treatment-naive)	Not reached
I/II [128]	Selpercatinib	RET	4	Salivary gland	50%	Not Reported	Not Reported
I/II [129]	Pralsetinib	RET	29	Various solid tumors (excluding NSCLC and thyroid)	57%	7 months	14 months
I/II [129]	Pralsetinib	RET	1	SGM	Not Reported	Not Reported	Not Reported

ORR: Overall response rate, MPFS: Median progression-free survival, MOS: Median overall survival in months, NSCLC: Non-small-cell carcinoma, AdCC: Adenoid cystic carcinoma.

**Table 12:** Efficacy of dabrafenib + trametinib in a clinical trial.

Phase	Agent	Target	Pts (n)	Subtype	Objective Response Rate	Median Progression-Free Survival (Months)	Median Overall Survival (Months)
II [138]	Dabrafenib + Trametinib	BRAF V600E + MEK1/2	36	BRAF-mutated solid tumors	41%	Not reported	Not reported

ORR: Overall response rate, MPFS: Median progression-free survival, MOS: Median overall survival in months.

**Table 13:** Efficacy of imatinib in adenoid cystic carcinoma in clinical trials.

Phase	Setting	Agent	Target	Pts, n	Subtype	Objective Response Rate	Median Progression-Free Survival (Months)	Median Overall Survival (Months)
II [141]	R/M Any line	Imatinib	c-kit, BCR-ABL, PDGFR	16	c-kit+ AdCC	0 (0%)	2.3	7
II [142]	R/M Any line	Imatinib	c-kit, BCR-ABL, PDGFR	10	AdCC	0 (0%)	6	Not reported
II [143]	R/M Any line	Imatinib + Cisplatin	Imatinib: c-kit, BCR-ABL, PDGFR Cisplatin: cytostatic drug	28	c-kit^+^ AdCC	3 (11%)	15	35

ORR: Overall response rate, MPFS: Median progression-free survival, MOS: Median overall survival in months, AdCC: Adenoid cystic carcinoma.

**Table 15:** Efficacy of PI3K/Akt inhibitors in SGMs in clinical trials.

Phase	Agent	Target	Pts (n)	Subtype	Objective Response Rate	Median Progression-Free Survival (Months)	Median Overall Survival (Months)
II [159]	Nelfinavir	Akt pathway	15	AdCC	0%	5.5	Not reported
II [160]	Everolimus	mTOR	34	AdCC	0%	11.2	23.7
II [164]	Bortezomib	NF-kB	21	AdCC	0%	6.4	21

ORR: Overall response rate, MPFS: Median progression-free survival, MOS: Median overall survival in months, AdCC: Adenoid cystic carcinoma.

## Data Availability

The original data presented in the study are openly available on PubMed, Google Scholar, and Embase databases.

## Abbreviations

SGC	Salivary Gland Carcinoma
HER2	Human Epidermal Growth Factor Receptor 2
NTRK	Neurotrophic Tropomyosin Receptor Kinase
RET	Rearranged during Transfection (a tyrosine kinase receptor)
AR	Androgen Receptor
EGFR	Epidermal Growth Factor Receptor
MAPK/ERK	Mitogen-Activated Protein Kinase/Extracellular Signal-Regulated Kinase pathway
PI3K/AKT	Phosphoinositide 3-Kinase/Protein Kinase B pathway
JAK/STAT	Janus Kinase/Signal Transducer and Activator of Transcription pathway
EMT	Epithelial-to-Mesenchymal Transition
REFCOR	The French National Network on Rare Head and Neck Tumors
IHC	Immunohistochemistry
ADT	Androgen Deprivation Therapy
DOR	Duration of Response
DFS	Disease-Free Survival
PFS	Progression-Free Survival
OS	Overall Survival
TRK	Tropomyosin Receptor Kinase
IMRT	Intensity-Modulated Radiotherapy
CRT	Chemoradiotherapy
FDA	Food and Drug Administration
VEGFR	Vascular Endothelial Growth Factor Receptor
PDGFR	Platelet-Derived Growth Factor Receptor
FGFR	Fibroblast Growth Factor Receptor
BRAF	A gene involved in cell growth regulation
MEK	Mitogen-Activated Protein Kinase Kinase
ALK	Anaplastic Lymphoma Kinase
NF-κB	Nuclear Factor Kappa B (a protein complex involved in immune response)
PLAG1	Pleomorphic Adenoma Gene 1
SMARCB1	SWI/SNF-Related Matrix-Associated Actin-Dependent Regulator of Chromatin Subfamily B Member 1
NSCLC	Non-Small Cell Lung Cancer
RTOG	Radiation Therapy Oncology Group
MPFS	Median Progression-Free Survival
MOS	Median Overall Survival
MEC	Mucoepidermoid Carcinoma
AdCC	Adenoid Cystic Carcinoma
ACC	Acinic Cell Carcinoma
SDC	Salivary Duct Carcinoma
PAC	Polymorphous Adenocarcinoma
EMC	Epithelial–Myoepithelial Carcinoma
